# Purification and Functional Characterisation of Rhiminopeptidase A, a Novel Aminopeptidase from the Venom of *Bitis gabonica rhinoceros*

**DOI:** 10.1371/journal.pntd.0000796

**Published:** 2010-08-10

**Authors:** Sakthivel Vaiyapuri, Simon C. Wagstaff, Kimberley A. Watson, Robert A. Harrison, Jonathan M. Gibbins, E. Gail Hutchinson

**Affiliations:** 1 School of Biological Sciences, University of Reading, Whiteknights, Reading, United Kingdom; 2 Alistair Reid Venom Research Unit, Liverpool School of Tropical Medicine, Pembroke Place, Liverpool, United Kingdom; 3 Blood Transfusion Research Group, King Saud University, Riyadh, Saudi Arabia; McGill University, Canada

## Abstract

**Background:**

Snake bite is a major neglected public health issue within poor communities living in the rural areas of several countries throughout the world. An estimated 2.5 million people are bitten by snakes each year and the cost and lack of efficacy of current anti-venom therapy, together with the lack of detailed knowledge about toxic components of venom and their modes of action, and the unavailability of treatments in rural areas mean that annually there are around 125,000 deaths worldwide. In order to develop cheaper and more effective therapeutics, the toxic components of snake venom and their modes of action need to be clearly understood. One particularly poorly understood component of snake venom is aminopeptidases. These are exo-metalloproteases, which, in mammals, are involved in important physiological functions such as the maintenance of blood pressure and brain function. Although aminopeptidase activities have been reported in some snake venoms, no detailed analysis of any individual snake venom aminopeptidases has been performed so far. As is the case for mammals, snake venom aminopeptidases may also play important roles in altering the physiological functions of victims during envenomation. In order to further understand this important group of snake venom enzymes we have isolated, functionally characterised and analysed the sequence-structure relationships of an aminopeptidase from the venom of the large, highly venomous West African gaboon viper, *Bitis gabonica rhinoceros.*

**Methodology and Principal Findings:**

The venom of *B. g. rhinoceros* was fractionated by size exclusion chromatography and fractions with aminopeptidase activities were isolated. Fractions with aminopeptidase activities showed a pure protein with a molecular weight of 150 kDa on SDS-PAGE. In the absence of calcium, this purified protein had broad aminopeptidase activities against acidic, basic and neutral amino acids but in the presence of calcium, it had only acidic aminopeptidase activity (APA). Together with the functional data, mass spectrometry analysis of the purified protein confirmed this as an aminopeptidase A and thus this has been named as rhiminopeptidase A. The complete gene sequence of rhiminopeptidase A was obtained by sequencing the PCR amplified aminopeptidase A gene from the venom gland cDNA of *B. g. rhinoceros*. The gene codes for a predicted protein of 955 amino acids (110 kDa), which contains the key amino acids necessary for functioning as an aminopeptidase A. A structural model of rhiminopeptidase A shows the structure to consist of 4 domains: an N-terminal saddle-shaped β domain, a mixed α and β catalytic domain, a β-sandwich domain and a C-terminal α helical domain.

**Conclusions:**

This study describes the discovery and characterisation of a novel aminopeptidase A from the venom of *B. g. rhinoceros* and highlights its potential biological importance. Similar to mammalian aminopeptidases, rhiminopeptidase A might be capable of playing roles in altering the blood pressure and brain function of victims. Furthermore, it could have additional effects on the biological functions of other host proteins by cleaving their N-terminal amino acids. This study points towards the importance of complete analysis of individual components of snake venom in order to develop effective therapies for snake bites.

## Author Summary

Snake bite is a major neglected public health issue causing an estimated 125,000 deaths each year, predominantly within poor communities living in rural areas of countries in South East Asia and Africa. Current treatments for snake bites are costly and have limited effectiveness, thus there is a need to develop novel therapeutics. In order to do this the toxic components of snake venom need to be clearly understood. Enzymes called aminopeptidases have been noticed in several snake venoms, but their functions have not been characterised. Related enzymes are also present in mammals, where they are involved in the maintenance of blood pressure and brain function. To further understand this important group of enzymes within snake venom we have purified and analysed the function and structure of an aminopeptidase from the venom of the West African gaboon viper. Our results suggest that this enzyme could also affect the maintenance of blood pressure and brain function in victims of snake bites. Along with other snake venom components, aminopeptidases might be a potential therapeutic target for developing novel treatments for snake bites.

## Introduction

A detailed understanding of the components of snake venom is important both for acquiring a more complete understanding of the pathology of envenoming and to aid in the development of improved treatments for snake bites, which are the cause of many deaths throughout the world each year. Snake venoms are complex mixtures of enzymatic and non enzymatic proteins, together with other components such as carbohydrates, lipids, nucleosides and metals. These function together to immobilize, kill and digest prey [1]. Some proteins such as hyaluronidase and L-amino acid oxidase are present in all 4 snake families (Viperidae, Atractaspididae, Elapidae and Colubridae), while other proteins are restricted to certain families. For example viper venom has predominantly hemorrhagic, hypotensive and inflammatory effects, caused by the metalloproteases, serine proteases and C-type lectins present, while neurotoxins, which affect the central nervous system, are major constituents of elapid snake venoms. Despite extensive studies on individual proteins and many recent proteomic and transcriptomic analyses of snake venoms [2] there remains much to be learned about the components of snake venom and their functions.

One of the least understood enzyme constituents of snake venoms is aminopeptidases. These enzymes remove one or more specific N-terminal residues from target proteins or peptides. For example aminopeptidase L (APL) removes an N-terminal leucine residue, aminopeptidase A (APA) removes an acidic N-terminal residue, aminopeptidase B (APB) removes a basic N-terminal residue, and aminopeptidase N (APN) removes a neutral N-terminal residue, typically alanine. There have been several reports of aminopeptidase activities present in venoms from elapids and vipers [3], [4], [5], [6], [7], [8], and a fraction exhibiting aminopeptidase A activity has been separated from the venom of *Gloydius blomhoffi brevicaudus*, a member of the Crotalinae (pit viper) subfamily of vipers [7]. A cDNA sequence from this snake represents the only determined sequence to date of a reptile venom aminopeptidase A. Interestingly, none of the complete snake venom proteomic studies done thus far has identified aminopeptidases [9]. Further study of such enzymes is important in order to understand their role within snake venom, and to help in the development of improved treatments for snake bite. Knowledge about this enzyme may also contribute to our knowledge of related mammalian enzymes such as mammalian APA, which is a candidate target for the treatment of hypertension.

Here we demonstrate aminopeptidase activity in the venom of *B. g. rhinoceros*, a member of the Viperinae (true viper) subfamily of vipers and report for the first time the complete purification of a snake venom aminopeptidase which we have named rhiminopeptidase A. We have functionally characterised this enzyme and obtained cDNA and amino acid sequences. Since structural information is lacking both for snake venom aminopeptidases and for their mammalian homologues, we have created a structural model for rhiminopeptidase A. This, together with the sequence, is consistent with the ability of this enzyme to function as a calcium-modulated aminopeptidase A and could inform efforts in the future to develop improved treatments both for snake bites and for hypertension.

## Materials and Methods

### Materials

Lyophilized venom of *B. g. rhinoceros* was obtained from an existing collection of pooled venom labelled ‘*Bitis gabonica* Nigeria Box 13/Bot 10’ which was stored at the Liverpool School of Tropical Medicine, Liverpool, UK (LSTM). Protein molecular weight markers and polyvinylidene fluoride (PVDF) membranes were from Bio-Rad. The low molecular weight column calibration kit, enhanced chemiluminescence (ECL) reagents and ECL glycoprotein detection module were from GE Healthcare. N Glycosidase F enzyme was from Roche Diagnostics Limited, and trypsin, thrombin and the GoTaq PCR Core System were from Promega. L-Glutamyl-7-amido-4-methylcoumarin (Glu-AMC) and L-aspartyl-7-amido-4-methylcoumarin (Asp-AMC) were obtained from Bachem. Macrosol and Stura crystallisation screening kits were from Molecular Dimensions Ltd and Wizard screening kits were obtained from Emerald BioSystems. L-Leucine-7-amido-4-methylcoumarin hydrochloride (Leu-AMC), L-Arginine-7-amido-4-methylcoumarin hydrochloride (Arg-AMC) and L-Alanine 7-amido-4-methylcoumarin trifluoroacetate salt (Ala-AMC) were obtained from Sigma-Aldrich. All other chemicals used were analytical grade from Sigma Aldrich.

### SDS-PAGE and immuno blotting

Reducing SDS-PAGE, gel staining and immunoblotting on to PVDF membrane were all performed using standard techniques [10].

### Protein purification

50 mg of *B. g. rhinoceros* venom were dissolved in 2 ml of 0.02 M Tris-HCl pH 7.4 and loaded on to a Sephacryl HR 200 gel filtration column. 31ml fractions were collected using 0.02 M Tris-HCl pH 7.4 at a speed of 1 ml/minute after 40 ml of pre-elution. 100 µl of selected fractions were analysed by 10% reducing SDS-PAGE. The purified protein was quantified using the Bradford method [11].

### Q-TOF analysis

This analysis was carried out at M-Scan Limited, Wokingham, UK. A band containing purified protein from a colloidal Coomassie stained 10% SDS-PAGE gel was sliced, reduced, alkylated and subjected to tryptic digestion. The resulting peptides were extracted and analysed by nano LC-ES-MS/MS using a Dionex Ultimate 3000 HPLC system coupled to a Q-TOF mass spectrometer. Data-dependent acquisition was utilised and peptides eluting from the nano LC column were automatically fragmented in the Q-TOF by recognition of their doubly or triply charged ion states. Preset charge and mass dependent collision voltages were applied by the software, which also allowed simultaneous MS/MS of up to 3 peptides. Processed spectral data were used to interrogate the mass spectrometry sequence database (MSDB) housed locally, using MASCOT software [12]. Several spectra were also checked manually in order to confirm automated peptide assignments. Glu-fibrinopeptide fragment ions in MS/MS mode were used to calibrate the instrument over the appropriate mass range.

### Functional characterisation

Aminopeptidase activities of venom and purified protein were measured using fluorescent substrates (Leu-AMC to measure APL activity, Arg-AMC to measure APB activity, Ala-AMC to measure APN activity and Glu-AMC and Asp-AMC for APA activity) as previously described [7], [10], [13]. Ten micrograms of venom or purified protein were mixed with various concentrations of substrate solutions and incubated at 37°C. Experiments were performed with and without 1.2 mM calcium chloride present. The amount of 7-amido-4-methylcoumarin (AMC) released was measured at different time intervals by spectrofluorimetry (FLUOstar OPTIMA, Offenburg, Germany) at an excitation wavelength of 366 nm and an emission wavelength of 460 nm. The kinetic parameters were calculated from Lineweaver-Burk plots. The results are represented by K_m_, k_cat_ and k_cat_/K_m_ values. All measurements were obtained from three separate experiments. To test the effect of various metal ions and protease inhibitors on aminopeptidase activity, the purified protein (10 µg) was pre-incubated with various concentrations of metal ions or inhibitors at 37°C for 5 minutes. Then, 50 nM of Glu-AMC was added to each sample and incubated for 10 minutes at 37°C. The amount of AMC liberated was measured as mentioned above.

### Glycosylation detection and deglycosylation

Twenty micrograms of native protein were subjected to (i) 10% reducing SDS-PAGE followed by transfer to a PVDF membrane and (ii) glycosylation detection using the ECL glycoprotein detection module according to the manufacturer's protocol. Deglycosylation was achieved by mixing 100 µg of purified protein in 0.02 M Tris-HCl pH 7.4 with 5 units of N Glycosidase F in a total volume of 50 µl and incubating for 10 hours at 37°C.

### cDNA amplification and sequencing

The cDNA of the *B. g. rhinoceros* venom gland was obtained from the cDNA library of *B. g. rhinoceros* (LZ7) which had been created for another study and was maintained at LSTM, Liverpool. Specific primers were designed based on the untranslated regions of the aminopeptidase A gene sequence from *G. b. brevicaudus* (NCBI accession number: AB262071) and synthesized by Sigma Aldrich, Poole, UK. The sequences of the primers are: forward primer - 5′CAAGCAAAAGCAGATGAGAAGGAA3′ and reverse primer - 5′TCAGAGTGGCGAATA TGTGGTTA3′. These were used to amplify the aminopeptidase A gene by PCR (25 cycles) using denaturation at 94°C for 30 seconds, annealing at 54°C for 30 seconds, extension at 72°C for 3.5 minutes and a final extension at 72°C for 10 minutes. The amplified product was analysed by 1% agarose gel electrophoresis and sequenced by Cogenics Limited, Essex, UK.

### Sequence analysis

The nucleotide sequence was translated and the molecular weight and estimated pI of the predicted protein were analysed using DNASTAR Lasergene software version 7 [14]. Similar sequences in the NCBI database were identified using BLAST. Multiple sequence alignments were performed with ClustalW2 [15] using default parameters of KTUP and gap opening and gap extension penalties. Transmembrane helices were predicted using PolyPhobius [16]. Interproscan [17] was used to identify functional domains within the sequence. Predicted N-Glycosylation sites were identified using the NetNGlyc 1.0 server (http://www.cbs.dtu.dk/services/NetNGlyc/ R. Gupta, E. Jung, S. Brunak manuscript in preparation).

### Crystallisation and data collection

Purified rhiminopeptidase A from *B. g. rhinoceros* venom (in 0.02 M Tris-HCl pH17.4) was concentrated to 9 mg/ml using centrifugal membrane concentrators. Initial crystallisation screening was performed manually in 2 plus 2 µl drops using the hanging drop vapour-diffusion method in 24-well Linbro plates against the following commercial screens at 18°C: Macrosol I and II, Stura Footprint Screen I and II and Wizard I and II. From the 288 conditions screened, three hits (one from each screen) were found showing small rod-like crystals. Crystals typically appeared between 7 and 14 days. The most promising condition was Macrosol I No. 9 [8% (w/v) PEG-3500, 0.1 M sodium acetate pH 4.5, 0.2 M ammonium acetate], which gave crystals with dimensions 50×20×10 µm. Micro-seeding was performed to increase the size and quality of the crystals obtained in screening. X-ray diffraction data were collected on an ADSC Q315 CCD detector at 100K on the macromolecular crystallography Beamline ID14-EH1 (ESRF, France). Integration and scaling of the diffraction data were performed using MOSFLM and SCALA, respectively [18], [19].

### Structural modelling of rhiminopeptidase A

Secondary structure prediction was performed using PSI-PRED [20]. BLAST, genTHREADER [21] and Phyre [22] were used to identify the best template structure to use for creating a structural model. The template selected was the X-ray crystallographic structure of tricorn interacting factor F3 from the archaeon *Thermoplasma acidophilum* (PDB code 1z5h) [23]. The alignment of rhiminopeptidase A with tricorn interacting factor F3 was determined using alignments obtained from mgenTHREADER and Phyre. Three-dimensional structural models were constructed using the MODELLER comparative protein structure modelling program [24]; these were evaluated using Procheck [25] and ModFOLD [26] and the best quality model selected. Models were visualised using PyMOL (DeLano Scientific).

## Results

### Purification of a 150 kDa protein from the venom of *B. g. rhinoceros*

**Figure 1:**
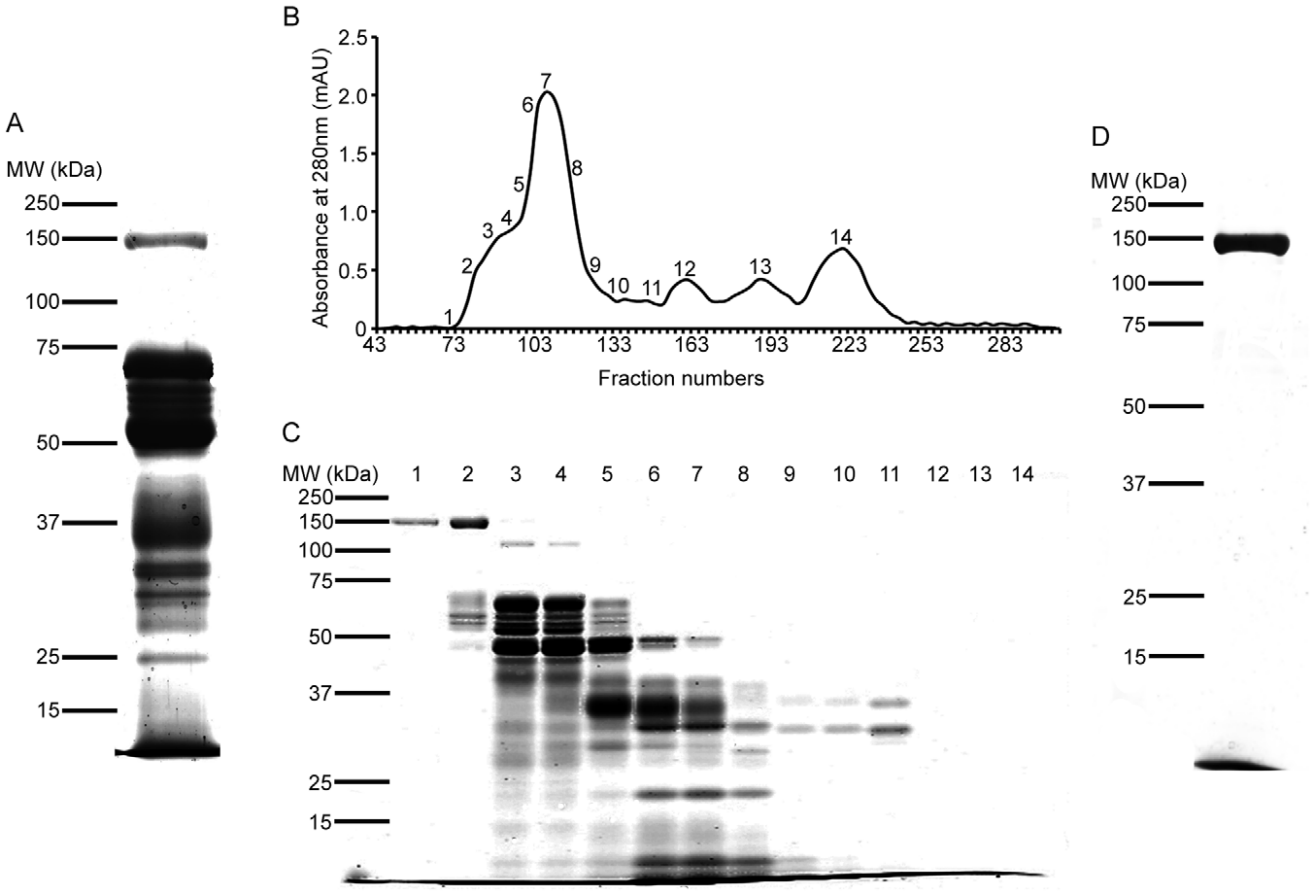
Purification of a 150 kDa protein from *Bitis gabonica rhinoceros* venom. *A,* SDS-PAGE of *B. g. rhinoceros* venom. 50 µg of venom were run on 10% SDS-PAGE and stained with Coomassie brilliant blue. A number of bands are present, including a prominent band with an approximate molecular mass of 150 kDa. *B*, Purification profile of *B. g. rhinoceros* venom using gel filtration. 50 mg of venom dissolved in 2 ml of 0.02 M Tris-HCl pH 7.4 were loaded on to a Sephacryl HR 200 gel filtration column. 3 ml fractions were collected using 0.02 M Tris-HCl pH 7.4 at a speed of 1 ml/minute after 40 ml of pre-elution. 14 fractions from the profile (labelled 1 to 14) were chosen for SDS-PAGE analysis. *C*, SDS-PAGE of the selected gel filtration fractions. 100 µl of each of fractions 1 to 14 were run on a 10% SDS-PAGE gel. Lane 1 (fraction 1) contains only a protein of molecular mass 150 kDa and lane 2 (fraction 2) contains this protein along with several others. *D*, SDS-PAGE of the purified 150 kDa protein. Fractions containing pure 150 kDa protein were pooled and concentrated using vivaspin filters and 100 µl were run on a 10% SDS-PAGE gel.

SDS-PAGE of whole *B. g. rhinoceros* venom (Fig. 1A) shows a number of bands including a prominent well-resolved band at an approximate molecular mass of 150 kDa, which is approximately what one might expect for an aminopeptidase, consistent with previously characterised aminopeptidases (120–185 kDa [27]). This venom was fractionated using a 1.6 cm×95 cm Sephacryl HR 200 gel filtration column (Fig. 1B) and 14 fractions were analysed by SDS-PAGE (Fig. 1C). A protein with molecular weight 150 kDa was found purified to apparent homogeneity on SDS-PAGE in fraction 1 (Fig. 1C) and partially purified in fraction 2. Two sub-fractions between fractions 1 and 2 also contained pure 150 kDa protein (data not shown). These 2 sub-fractions, together with fraction 1, were pooled and concentrated by ultrafiltration in order to obtain the maximum amount of pure 150 kDa protein (Fig. 1D). Using the Bradford assay [11] the estimated amount of protein obtained from 50 mg of whole venom was 1.3 mg.

### Identification of a 150 kDa protein by mass spectrometry

Sequence information was obtained by nano LC-ES-MS/MS of peptides derived by tryptic digestion of the 150 kDa gel band. Interrogation of the mass spectrometry database (MSDB) with the MS and MS/MS peak lists using MASCOT software identified a hypothetical protein from *Pongo pygmaeus* (Bornean orang-utan; MSDB accession number Q5R7D5_PONPY) (P-value = 5×10^−18^) as the only non-contaminant protein. The sequence of this protein was 100% identical to that of a glutamyl aminopeptidase from *Pongo abelii* (Sumatran orang-utan) found in the NCBI sequence database (accession number NP_001126365.1). Manual sequencing of an individual MS/MS spectrum yielded the following sequence: GFI/LDDAFAI/LAR. Protein-Protein BLAST using the sequence GFIDDAFALAR showed that it was 100% identical to a fragment of aminopeptidase A from *G. b. brevicaudus* [7]. These results, together with the estimated molecular mass, suggest that the 150 kDa protein might be an aminopeptidase.

### Functional characterisation of the 150 kDa protein

**Figure 2:**
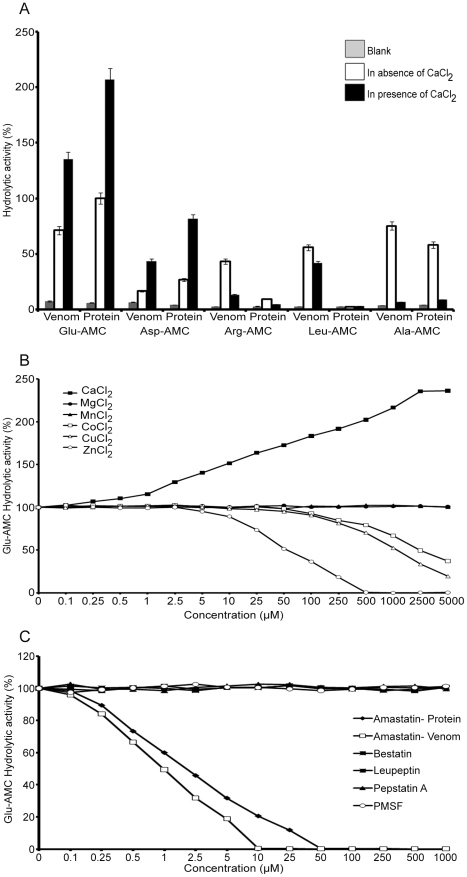
Functional characterisation of the 150 kDa protein. *A*, Functional assays for aminopeptidase activities [aminopeptidase A (APA), aminopeptidase B (APB), leucine aminopeptidase (APL) and neutral aminopeptidase (APN)] were performed on the purified protein and on the whole *B. g. rhinoceros* venom. 10 µg of venom or purified protein were mixed with 50 nM of fluorescent substrates (Leu-AMC to measure APL activity, Arg-AMC to measure APB activity, Ala-AMC to measure APN activity and Glu-AMC and Asp-AMC to measure APA activity) and the final reaction volume was made up to 100 µl with 0.02 M Tris.HCl pH 7.4, and the reaction mix was incubated for 10 minutes at 37°C. Experiments were performed in the absence and presence of 1.2 mM calcium chloride. The amount of 7-amido-4-methylcoumarin (AMC) released was measured by spectrofluorimetry at an excitation wavelength of 366 nm and emission wavelength of 460 nm. The data represent the mean ± S.D. (*n* = 3 separate experiments). The hydrolytic activity measured for the 150 kDa protein against Glu-AMC in the absence of 1.2 mM CaCl_2_ was taken as 100%. *B*, The effects of various divalent cations on the enzymatic activity of rhiminopeptidase A. 10 µg of purified protein were pre-incubated with various concentrations of divalent cations for 5 minutes before the addition of 50 nM Glu-AMC. The reaction was incubated for 10 minutes at 37°C and the liberated AMC was measured. The hydrolytic activity in the absence of metal ions was taken as 100%. The data are representative of three separate experiments. *C*, The effect of various protease inhibitors on the enzymatic activity of rhiminopeptidase A. 10 µg of purified protein were pre-incubated with various concentrations of protease inhibitors for 5 minutes before the addition of 50 nM Glu-AMC. Similarly 10 µg of *B. g. rhinoceros* whole venom were pre-incubated with various concentrations of amastatin for 5 minutes before the addition of 50 nM Glu-AMC. The reaction was incubated for 10 minutes at 37°C and the liberated AMC was measured. The hydrolytic activity in the absence of metal ions was taken as 100%. The data are representative of three separate experiments.

To further investigate the function of the 150 kDa protein, functional assays for the main aminopeptidase activities (APA, APB, APL and APN) were performed on the whole *B. g. rhinoceros* venom and on the purified protein using fluorescent substrates as previously described [7], [13] (Fig. 2A). The venom showed significant levels of all the aminopeptidase activities tested, with APN>APA (Glu-AMC)>APL>APB>APA (Asp-AMC), while the 150 kDa protein displayed relatively high APA (Glu-AMC) and APN activities, moderate APA activity towards Asp-AMC, very low APB and negligible APL activities. In the presence of calcium chloride the APA activities of both the venom and the pure protein towards both Glu-AMC and Asp-AMC increased substantially (by at least 90% for Glu-AMC and more than 160% for Asp-AMC activity), while all other aminopeptidase activities of the protein were negligible, suggesting that in the presence of calcium the enzyme shows increased specificity towards acidic amino acids. A calcium titration showed that the highest aminopeptidase activity towards Glu-AMC was obtained using 1.2 mM calcium chloride (data not shown). In the presence of calcium the APN activity of the venom was also negligible, but the APL activity was reduced by 25% and some APB activity remained. The observed activities of the protein were consistent with the partial sequence identification and provide further evidence that the 150 kDa protein is an aminopeptidase A. Thus we have named this protein ‘rhiminopeptidase A’. The detection of APL and APB activities in the whole venom both with and without calcium present suggests the presence of one or more further aminopeptidases in the venom of *B. g. rhinoceros*.

Table 1 shows the enzymatic parameters of rhiminopeptidase A measured in the presence and absence of 1.2 mM CaCl_2_. In the presence of Ca^2+^ ions, the hydrolytic activity of rhiminopeptidase A was enhanced by increasing the k_cat_ value and decreasing the K_m_ value. However, these data confirm that the enzyme is more active against Glu-AMC than Asp-AMC. Fig. 2B shows the effects of various divalent cations on the activity of rhiminopeptidase A. When rhiminopeptidase A was incubated with Glu-AMC in the presence of Ca^2+^ ions, the hydrolytic activity increased. However, in the presence of Zn^2+^ ions the hydrolytic activity was strongly reduced, and eliminated completely at 0.5 mM. Co^2+^ and Cu^2+^ ions showed inhibition at higher concentrations and Mn^2+^ and Mg^2+^ ions showed no inhibitory effects on rhiminopeptidase A activity against Glu-AMC. These data suggest that Ca^2+^ is the only divalent cation to enhance the hydrolytic activity of rhiminopeptidase A towards Glu-AMC and that Zn^2+^ is the strongest inhibitor.

**Table 1:** Kinetic parameters of rhiminopeptidase A towards Glu-AMC and Asp-AMC.

Substrate	CaCl_2_ *1.2 mM*	K_m_ a *µM*	k_cat_ a *s ^−1^*	k_cat_/K_m_×10^3a^ *µM ^−1^s ^−1^*
Glu-AMC	−	2235±89	25.2±1.9	11.3±0.8
	+	703±25	63.1±2.7	89.7±1.2
Asp-AMC	−	4119±135	5.3±0.8	1.3±0.5
	+	2478±61	23.1±1.4	9.3±1.7

a The values are mean ± S.D. (*n* = 3).

Kinetic parameters were determined from Lineweaver-Burk plots. 10 µg of rhiminopeptidase A were incubated with various concentrations of Glu-AMC and Asp-AMC at 37°C for 7 minutes in the presence and absence of 1.2 mM CaCl_2_. The liberated AMC was measured at different time intervals up to 7 minutes using spectrofluorimetry at an excitation wavelength of 366 nm and an emission wavelength of 460 nm.

To analyse the effects of various protease inhibitors, rhiminopeptidase A was treated with amastatin (APL and APA inhibitor), bestatin (APL inhibitor), leupeptin (serine/cysteine protease inhibitor), pepstatin A (aspartic acid protease inhibitor) and PMSF (serine protease inhibitor) followed by incubating with Glu-AMC. As for known mammalian aminopeptidases, amastatin inhibited the Glu-AMC activity of rhiminopeptidase at a concentration of 50 µM (Fig. 2C), however, the other inhibitors had negligible effect on this activity. This confirms that the Glu-AMC activity of the purified rhiminopeptidase A is due to the activity of an aminopeptidase A and is not caused by any other enzyme. Furthermore, 10 µM amastatin inhibited completely the Glu-AMC activity of 10 µg of venom, confirming that the Glu-AMC activity observed in the snake venom comes exclusively from aminopeptidase A.

### Rhiminopeptidase A is a glycosylated protein

**Figure 3:**
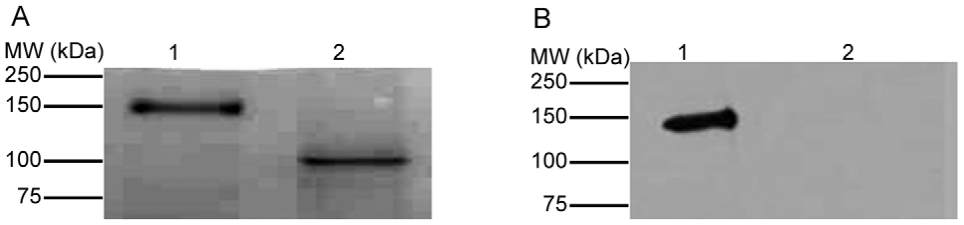
Rhiminopeptidase A is a glycosylated protein. The glycosylation of rhiminopeptidase A was detected using the ECL glycosylation detection module and deglycosylation was performed by mixing 100 µl of rhiminopeptidase A with 5 units of N glycosidase F in 50 µl of 0.02 M Tris-HCl pH 7.4. 20 µl of native (lane 1) and deglycosylated (lane 2) rhiminopeptidase A were run in two separate 10% SDS-PAGE gels and one of these was transferred to a PVDF membrane. *A,* the SDS-PAGE gel was stained with Coomassie Brilliant blue. *B*, the PVDF membrane was used to detect glycosylation using the ECL glycosylation detection module. Data are representative of three separate experiments.

As many snake venom enzymes are known to be glycosylated [28] and the aminopeptidase A from *G. b. brevicaudus* venom was predicted to be glycosylated [7], glycosylation detection was performed on rhiminopeptidase A using an ECL glycosylation detection module on a PVDF membrane. Rhiminopeptidase A was found to be substantially glycosylated. Thus deglycosylation was performed on the enzyme using N Glycosidase F and the resulting samples were run in 10% SDS-PAGE along with native rhiminopeptidase A. Fig. 3A shows that the estimated molecular mass of the deglycosylated protein was approximately 102 kDa, thus around 48 kDa (32%) of the molecular mass of the native purified protein is due to glycosylation. Another replicate gel was transferred to a PVDF membrane and subjected to glycosylation detection using the ECL glycosylation detection module. The lack of signal on the deglycosylated protein confirms the deglycosylation, while a signal was detected in the lane corresponding to the native rhiminopeptidase A (Fig. 3B).

### cDNA amplification and sequencing of rhiminopeptidase A

In order to obtain the complete sequence of rhiminopeptidase A cDNA was obtained from the stored venom gland cDNA library of a single *B. g. rhinoceros* snake (LZ7). Primers were designed based on the untranslated region of the *G. b. brevicaudus* aminopeptidase A gene [7]. PCR with these primers resulted in a product of approximately 3350 bp, which was consistent with the expected size of the aminopeptidase A gene.

**Figure 4:**
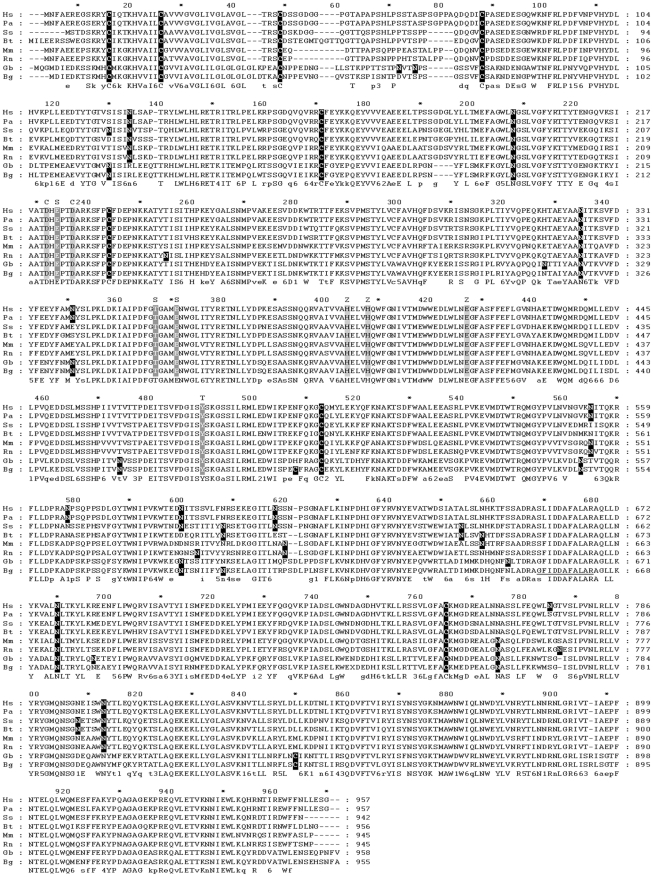
Multiple sequence alignment of rhiminopeptidase A with other aminopeptidase A sequences. The amino acid sequence of rhiminopeptidase A (Bg) was aligned with aminopeptidase A sequences from human (Hs, NP_001968), orang-utan (Pa, NP_001126365), pig (Ss, NP_999182), cow (Bt, NP_001033116), mouse (Mm, NP_031960), rat (Rn, AAF66704) and *G. b. brevicaudus* (Gb, BAF63164) using ClustalW2. Sequence conservation at each position is indicated by the letters underneath each section of the alignment: upper case letters indicate that the amino acid at that position is completely conserved and lower case letters indicate partial conservation. Cysteine residues and predicted N-glycosylation sites (predicted using NetNGlyc) are in white text with black background. Key functional residues based on similarity with the mouse sequence are indicated in bold font: zinc and calcium binding residues are in black type with grey shading and indicated by Z and C at the top of the column respectively; residues involved in substrate specificity and transition state stabilization are in white text with grey background and indicated by S and T at the top of the column respectively.

The nucleotide sequence of the amplified product contains 3232 nucleotides with an open reading frame between bases 66 and 2945 which encodes a polypeptide of 955 amino acids with an estimated molecular mass of 110.5 kDa and a predicted isoelectric point of 6.08. The latter coincides with the isoelectric point (6.2) of the native protein in the venom as determined by liquid phase isoelectric focussing (data not shown). Comparison of computer generated tryptic digested peptides derived from this amino acid sequence with the MS/MS data from the purified protein showed matches which covered 45% of the amino acid sequence, strongly suggesting that the sequence corresponds to the protein we have purified (Figure S1). Further, the partial sequence obtained from mass spectrometry is identical to the region of the sequence between amino acids 653 and 663 (underlined in Fig. 4). The absence of any other proteins with molecular weights around 150 kDa in the venom of *B. g. rhinoceros* or any other isoforms in the PCR amplified products provides further evidence that the gene we have sequenced corresponds to the rhiminopeptidase A protein which we had purified. The nucleotide sequence for the rhiminopeptidase A gene has been deposited in the EMBL database under Accession Number FN666431.

The protein sequence is 93% identical to that of aminopeptidase A from *G. b. brevicaudus*; 60–64% identical to aminopeptidase As from pig, cow, human, rat, mouse and orang-utan (Fig. 4); and 63–64% identical to predicted aminopeptidase As from horse, chimpanzee, Rhesus macaque, dog, platypus, opossum, chicken and Zebra finch. Consistent with our experimental identification of glycosylated moieties attached to rhiminopeptidase A, ten potential N-glycosylation sites were predicted in the protein sequence. Nine of these are shared with the *G. b. brevicaudus* APA sequence and three are conserved in all the sequences in the alignment.

There are 10 cysteine residues in the rhiminopeptidase A sequence, of which 8 are conserved in all the sequences compared. PolyPhobius [16] predicts a single transmembrane segment close to the start of the sequence, which is characteristic of a type II integral membrane protein. The recently cloned sequences of *G b. brevicaudus* APA and DPP IV were also predicted to be type II integral membrane proteins [7], [29] and exosome-like vesicles containing these proteins were subsequently detected in the *G. b. brevicaudus* venom [30].

Analysis using InterProScan [17] suggests that the protein is a zinc metallopeptidase belonging to MEROPS peptidase clan MA(E) (“gluzincins”) family M1. Gluzincin aminopeptidases are characterised by a consensus zinc binding motif HEXXHX_18_E [31], of which the two histidines and the final glutamic acid have been shown to act as the zinc ligands [32], [33], and a conserved GAMEN motif [34]. These motifs are conserved in rhiminopeptidase A and its relatives (Fig. 4). The alignment also shows conservation of several key functional residues: Glu^352^ within the GAMEN motif and Glu^215^,which have been shown to interact with the N-terminal amine of the substrate during catalysis [35], [36]; Thr^348^ which is involved in substrate specificity [37]; and Tyr^471^,which is involved in the stabilizing tetrahedral intermediate of the substrate during catalysis [38]. For consistency with other literature, the sequence numbering used here is for the mouse APA sequence (NCBI accession number NP_031960). Thus the sequence of rhiminopeptidase A contains the key amino acids required for it to function as a calcium-modulated aminopeptidase A.

### Structure determination by X-ray diffraction

Crystallisation trials have so far yielded only small crystals of rhiminopeptidase A. However, the crystals obtained were cryoprotected in mother liquor containing 25% (w/v) glycerol by quick transfer directly from the hanging drop and X-ray diffraction data were collected. Data analysis revealed a final resolution of 7.5 Å (Table 2). The crystals were of sufficient quality to show that the protein crystallised in the monoclinic space group P2_1_ with unit cell dimensions a = 97.6, b = 67.3, c = 186.6 Å, and β = 101.9° under the following conditions; 8% (w/v) PEG-3500, 0.1 M sodium acetate pH 4.5, 0.2 M ammonium acetate. Solvent content analysis using the programme MATTHEWS_COEF [39] suggested a solvent content of 30% with two molecules in the asymmetric unit.

**Table 2:** Data processing statistics for rhiminopeptidase A.

Synchrotron beamline, wavelength (Å)	ESRF ID14-EH1, 0.934
Space group	P2_1_
Unit cell dimensions (Å)	a = 97.6, b = 67.3, c = 186.6, β = 101.9°
Resolution range (Å)	67.27–7.50 (7.91–7.50)
R_merge_ †	0.113 (0.325)
No. of observations	7507 (1122)
No. of unique reflections	3053 (434)
Mean I/σ(I) ‡	8.5 (3.4)
Completeness (%)	96.0 (97.5)
Multiplicity	2.5 (2.6)
Solvent content (%)	29.0
Molecules per AU	2

**†:** R_merge_ = Σ*_h_* Σ_i_ | *I_h,i_* −<*I*_h_> |/Σ*_h_* Σ_i_ | *I_h,i_* |, where the outer summation is over all unique reflections with multiple observations and the inner summation is over all observations of each reflection.

**‡:** σ(I) is the standard deviation of *I*.

Values in parentheses correspond to the highest resolution shell (7.91–7.50 Å).

### Structure prediction

In the absence of an X-ray crystal structure we employed structure prediction tools to obtain a structural model for the rhiminopeptidase A protein. Secondary structure prediction using PsiPred 2.6 [20] and the secondary structure prediction tools used by Phyre [22] suggest that the protein has both α-helical and β-sheet regions, with the N-terminal regions being predominantly β-sheet and the C-terminal region being predominantly α-helical.

Using BLAST the most similar protein with a known structure is tricorn interacting factor F3 from the archaeon *Thermoplasma acidophilum* (PDB code 1z5h, [23]), which shares 31% sequence identity with rhiminopeptidase A. Tricorn interacting factor F3 is an 89 kDa zinc aminopeptidase with a strong preference for glutamate at the P1 position of the substrate [40] and is involved in the proteasomal degradation pathway of *T. acidophilum*. This structure was also confidently and consistently selected as the best template for modelling rhiminopeptidase A by several fold recognition servers [Phyre [22] (e-value = 0.0), mgenTHREADER [21] (p<0.0001)]. Structural models of rhiminopeptidase A were created using MODELLER software [24] and the best model was selected based on the scores obtained using ModFold [26] and Procheck [25].

**Figure 5:**
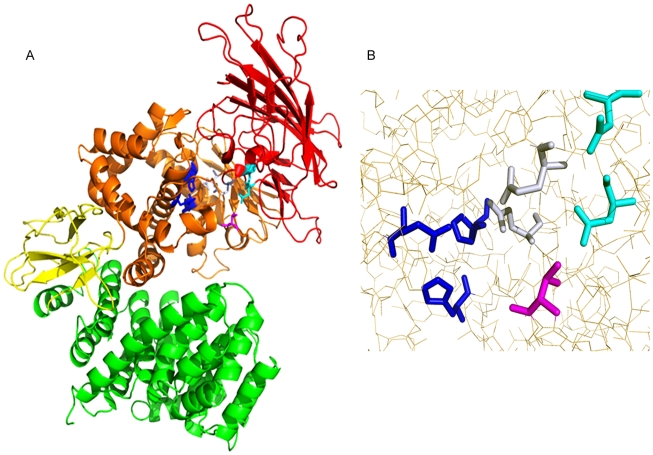
The modelled structure of rhiminopeptidase A. MODELLER was used to create a model of the structure of rhiminopeptidase A using the determined structure of tricorn interacting factor F3 from *T. acidophilum* (PDB code 1z5h) as a template. *A*, image of the 4-domain structure of rhiminopeptidase A with the domains coloured as follows: N-terminal saddle shaped β-sheet domain in red; catalytic domain in orange; β-sandwich domain in yellow and C-terminal α-helical domain in green. Key functional residues are highlighted as follows: zinc ligands in blue, calcium binding site in cyan, substrate binding residues in grey and the threonine involved in substrate specificity in magenta. *B*, detailed view of the key functional residues coloured as in A. The images were generated using PyMOL.

The model (Fig. 5A) includes residues 95 to 944 of the amino acid sequence and the predicted structure is very similar to that of tricorn interacting factor F3 (r.m.s.d. = 0.35 Å). Like F3 the predicted structure consists of 4 domains which together form a hook-like structure: an amino terminal saddle-shaped β-sheet domain, a mixed α and β catalytic domain, a β-sandwich domain and a large C-terminal α-helical domain. The sequences of the two proteins are most similar (46% identity) in the catalytic domain. Within the catalytic domain the three proposed zinc-binding residues in rhiminopeptidase A align with identical residues in the F3 sequence; these are in identical positions and orientations in the modelled rhiminopeptidase A structure and that of F3 and thus positioned appropriately to bind zinc (Fig. 5B). The residues proposed to be involved in substrate binding, calcium binding and substrate specificity are also in nearly identical positions in both structures. The final domain of F3 was found to be very flexible, and crystal structures were determined with the C-terminal portion of this in three different conformations which may relate to the structural changes which occur during substrate binding. Our structure was modelled based on the most open of these conformations but whether rhiminopeptidase A shares the flexibility of F3 in this region remains to be determined. The model co-ordinates have been deposited in the PMDB under accession number PM0076268.

Attempts were made to use the derived model (in its entirety and by individual domain) in a molecular replacement strategy to obtain a crystallographic structure solution using the current diffraction data. These have proved unsuccessful, likely owing to the low resolution limits of the current data and possibly due to the flexibility of the protein itself. Further efforts are underway to produce better diffraction quality crystals.

## Discussion

The data presented here demonstrate for the first time the presence of aminopeptidase activity in the venom of *B. g. rhinoceros*. We have purified an aminopeptidase A from this venom and shown that it has a relatively broad specificity (APN, APB and APA activities) in the absence of calcium, but a higher and very specific APA activity in the presence of calcium. This is consistent with the known calcium modulation of APAs [7], [41], [42], [43], [44]. As is the case for human aminopeptidase A, zinc ions act as an effective inhibitor of the APA activity of rhiminopeptidase A and copper and cobalt ions have a moderate inhibitory effect [44]. The aminopeptidase activity of the *B. g. rhinoceros* venom as a whole is different from that of the purified protein; the detection of APL and APB activity even in the presence of calcium suggests the presence of further aminopeptidases in this venom. APL and APB activities have been reported in the venoms of several other snakes [4], [6], [7], [8] but to date no-one has identified the specific enzymes responsible.

It is noteworthy that neither this protein nor any proteins which would have the APL or APB activities observed in the *B. g. rhinoceros* venom have been identified by proteomic studies of this venom [9]. There is also no reference to any aminopeptidases in the catalogue of transcripts encoded by the *B. gabonica* venom glands [45]. One possible reason for these discrepancies is that the approaches used for large scale identification of proteins or genes may make it difficult to detect low abundance, high molecular mass glycosylated proteins such as rhiminopeptidase A. An alternative reason for these differences could be variation of venom composition between individual snakes. Although we purified rhiminopeptidase A from pooled venom sourced from a number of snakes, we also ran gels on venom from seven individual snakes and showed that the protein profiles of the venoms from individual snakes were indistinguishable in terms of SDS-PAGE profiles both from each other and from the pooled venom. Thus the protein is likely to be present in at least the seven snakes which we analysed. It is clear that both large scale analyses and studies such as ours which focus on individual proteins remain important if we are to understand the complete spectrum of proteins present in snake venoms.

The amino acid sequence of rhiminopeptidase A contains the key amino acids which are known to be involved in aminopeptidase enzymatic function. APAs are the only M1 aminopeptidases which are modulated by calcium [46], and rhiminopeptidase A contains the two aspartic acid residues (Asp^216^ and Asp^221^; corresponding to Asp^213^ and Asp^218^ in mouse APA) which are thought to bind calcium. It also contains the amino acids which are thought to be important for the substrate specificity of APAs (Glu^218^, Glu^355^ and the recently established Thr^351^ [37], corresponding to Glu^215^, Glu^352^ and Thr^348^ in mouse APA). Interestingly, just prior to that study, the amino acid in that position had been suggested to be involved in the substrate specificity of aminopeptidases in general, and three subclasses of exopeptidases had been proposed: containing MGAMEN, AGAMEN and F/YGAMEN motifs [47]. The methionine has been proposed to exist in enzymes with broad specificities and to act as a cushion to accept substrates with different N-terminal sizes [48], LTA4H, which contains the F/YGAMEN motif prefers basic or aromatic residues and AGAMEN is found in F3, which prefers acidic residues. Rhiminopeptidase A and its homologues are also specific for acidic residues, but contain a TGAMEN motif, which may constitute an extension of the AGAMEN subclass. We have also found proteins with SGAMEN and PGAMEN sequences in the Uniprot database, although the correlation between the residue directly before the GAMEN motif and the sequence specificity becomes less clear when a wider range of sequences is considered.

Given the importance of M1 peptidases in many organisms, it is important to obtain an understanding of their structures. Structural information is currently limited: to date only the structures of human LTA4H [49], *T. acidophilum* F3 [23] and aminopeptidase Ns from *Neisseria meningitidis* [50], *Escherichia. coli* [47], [48] and *Plasmodium falciparum* [51]are known. These proteins have low sequence identities, although their structures are well conserved, particularly in the catalytic region. Information about the remaining domains is more variable. When the F3 structure was determined, the β-sandwich domain was thought to be unique to this protein, as it had not been found in the structure of LTA4H. However the aminopeptidase structures from *N. meningitidis*, *E. coli* and *P. falciparum* also have a β-sandwich domain, so this is no longer a unique feature of F3. We have selected the F3 structure as the best template for creating a model of rhiminopeptidase A and our model structure also has this domain. This is consistent with the results of two secondary structure prediction programs which confidently predict this region of the protein to be exclusively β-sheet. This may have implications for the structures of other M1 peptidases. For example a model of mouse aminopeptidase [52] was created using the LTA4H structure as a template prior to the availability of the F3 structure and lacks this domain. The roles of the domains other than the catalytic domain are unclear, though their interaction with the catalytic domain clearly affects the substrates which can bind to the enzyme, and one study has suggested the role of other regions of the protein in interacting with other proteins [53]. The C-terminal region of mouse aminopeptidase A (which corresponds to the final domain and around one third of the β-sheet domain) has been shown to act as an intramolecular chaperone, being required for the correct folding of the enzyme but not for the enzymatic activity [54].

The potential roles of aminopeptidases in snake venom are far from clear. Indeed, although aminopeptidases are expressed in many mammalian tissues, even their roles are not completely understood. Generally, mammalian aminopeptidases have been found to cleave oligopeptides. For example mammalian APA cleaves brain angiotensin II to yield angiotensin III, and is thus implicated in the control of arterial blood pressure [55]. *In vivo* APA has also been shown to cleave cholecystokinin (CCK-8) [56], which is widely distributed in the mammalian central nervous system and could be involved in pain perception, feeding, anxiety and memory. Other possible natural substrates which have only been tested *in vitro* include neurokinin B, chromogranin and kallidin [44]. The latter lacks an acidic N-terminal amino acid, and is converted to bradykinin only in the absence of calcium. Together these results support the idea that mammalian APA is important for regulation of brain function, and blood pressure in particular, but further substrates may yet be found. Some studies suggest a role for APA in blood vessel formation, and these could reflect a more general effect of APA on angiogenic mechanisms such as a role in degrading the extracellular matrix [57]. Ogawa *et al.* [7] have shown that exosome-like vesicles isolated from *G. b. brevicaudus* venom contain APA and, like mammalian APA, degrade both angiotensin II and CCK-8. It is therefore possible that a role of snake venom aminopeptidases is to cleave the N-termini of such oligopeptides in the victim and thus affect the corresponding physiological processes. Alternatively the aminopeptidases may simply assist the general degradation of the host tissue [3], perhaps increasing its permeability to other venom components [8]. A further possible role for snake venom aminopeptidases could be to process other toxins within the venom [8] and it is entirely possible that the enzymes have more than one of these suggested roles. The diversity and relatively high levels of aminopeptidase in snake venoms offer a valuable source of protein for characterisation of this complex family of enzymes. As this is an important group of venom enzymes which may be involved in critical envenomation effects in victims of snake bite, these enzymes could be potential therapeutic targets for developing novel snake bite treatments. This study clearly points towards the importance of complete analysis of individual components of snake venom in order to develop effective therapies for snake bites.

## Supporting Information

Figure S1 Comparison of computer generated tryptic digested peptides derived from the rhiminopeptidase A amino acid sequence with the MS/MS data from the purified protein. The figure shows the rhiminopeptidase A sequence with peptides matching the MS/MS data shown in bold red. The matched peptides cover 45% of the amino acid sequence, strongly suggesting that the sequence corresponds to the protein we have purified.
(0.86 MB TIF)
