# Supplementary figures and images for: Purification and Functional Characterisation of Rhiminopeptidase A, a Novel Aminopeptidase from the Venom of Bitis gabonica rhinoceros
Source: PLoS Negl Trop Dis. 2010 Aug 10;4(8):e796. doi: 10.1371/journal.pntd.0000796 (PMC2919393; doi:10.1371/journal.pntd.0000796)

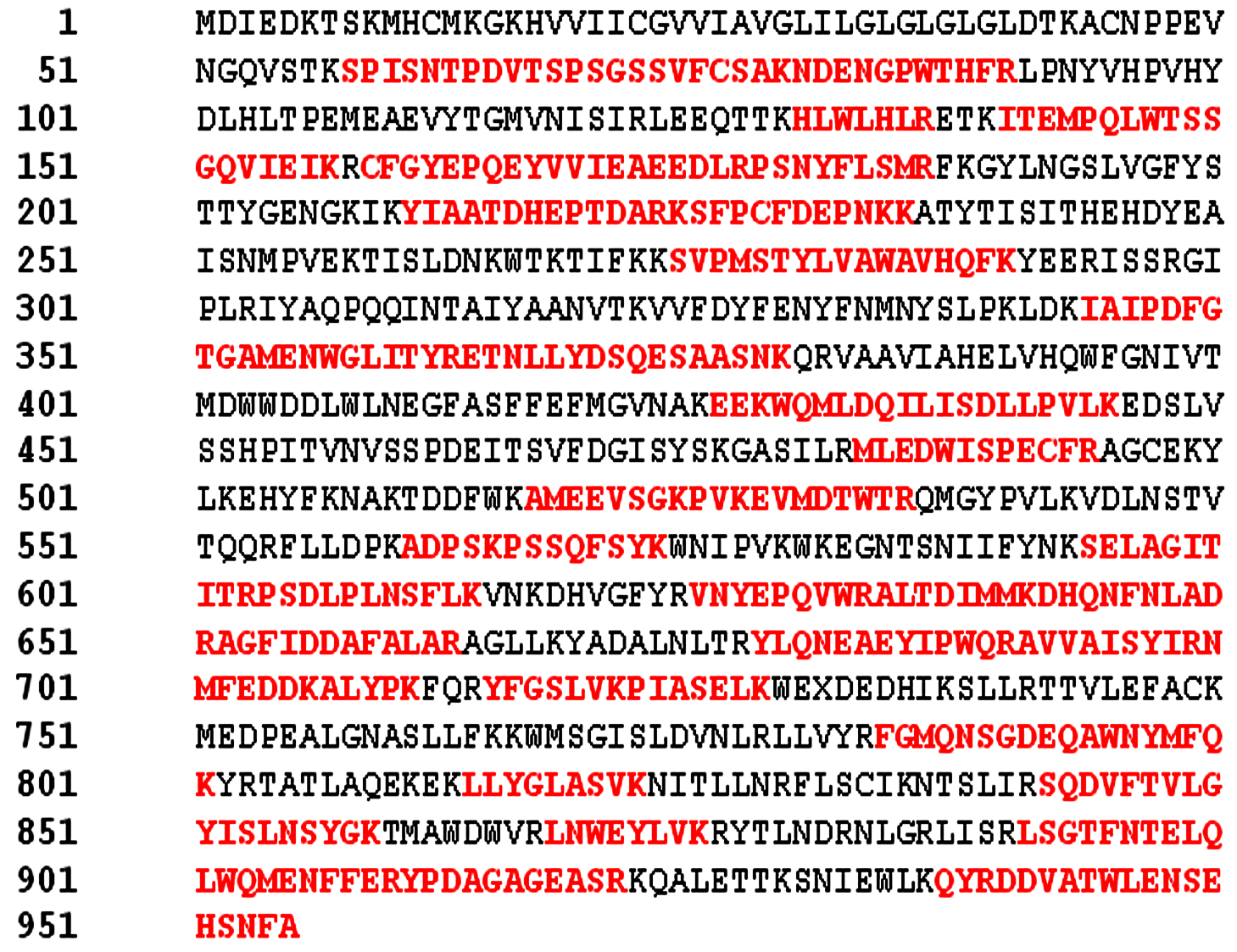

Supplement: Figure S1 — Comparison of computer generated tryptic digested peptides derived from the rhiminopeptidase A amino acid sequence with the MS/MS data from the purified protein. The figure shows the rhiminopeptidase A sequence with peptides matching the MS/MS data shown in bold red. The matched peptides cover 45% of the amino acid sequence, strongly suggesting that the sequence corresponds to the protein we have purified. (0.86 MB TIF) [file pntd.0000796.s001.tif]
